# Untargeted Metabolomics Reveals Species-Specific Metabolite Production and Shared Nutrient Consumption by Pseudomonas aeruginosa and Staphylococcus aureus

**DOI:** 10.1128/mSystems.00480-21

**Published:** 2021-06-22

**Authors:** Laura J. Dunphy, Kassandra L. Grimes, Nishikant Wase, Glynis L. Kolling, Jason A. Papin

**Affiliations:** a Department of Biomedical Engineering, University of Virginia, Charlottesville, Virginia, USA; b Department of Engineering Systems and Environment, University of Virginia, Charlottesville, Virginia, USA; c Biomolecular Analysis Facility, School of Medicine, University of Virginia, Charlottesville, Virginia, USA; d Division of Infectious Diseases and International Health, Department of Medicine, University of Virginia, Charlottesville, Virginia, USA; e Department of Biochemistry and Molecular Genetics, University of Virginia, Charlottesville, Virginia, USA; University of Massachusetts Medical School

**Keywords:** LC-MS, metabolism, *Pseudomonas aeruginosa*, *Staphylococcus aureus*

## Abstract

While bacterial metabolism is known to impact antibiotic efficacy and virulence, the metabolic capacities of individual microbes in cystic fibrosis lung infections are difficult to disentangle from sputum samples. Here, we show that untargeted metabolomic profiling of supernatants of multiple strains of Pseudomonas aeruginosa and Staphylococcus aureus grown in monoculture in synthetic cystic fibrosis media (SCFM) reveals distinct species-specific metabolic signatures despite intraspecies metabolic variability. We identify a set of 15 metabolites that were significantly consumed by both P. aeruginosa and S. aureus, suggesting that nutrient competition has the potential to impact community dynamics even in the absence of other pathogen-pathogen interactions. Finally, metabolites that were uniquely produced by one species or the other were identified. Specifically, the virulence factor precursor anthranilic acid, as well as the quinoline 2,4-quinolinediol (DHQ), were robustly produced across all tested strains of P. aeruginosa. Through the direct comparison of the extracellular metabolism of P. aeruginosa and S. aureus in a physiologically relevant environment, this work provides insight toward the potential for metabolic interactions *in vivo* and supports the development of species-specific diagnostic markers of infection.

**IMPORTANCE** Interactions between P. aeruginosa and S. aureus can impact pathogenicity and antimicrobial efficacy. In this study, we aim to better understand the potential for metabolic interactions between P. aeruginosa and S. aureus in an environment resembling the cystic fibrosis lung. We find that S. aureus and P. aeruginosa consume many of the same nutrients, suggesting that metabolic competition may play an important role in community dynamics during coinfection. We further identify metabolites uniquely produced by either organism with the potential to be developed into species-specific biomarkers of infection in the cystic fibrosis lung.

## INTRODUCTION

Pseudomonas aeruginosa and Staphylococcus aureus are opportunistic pathogens that commonly infect the lungs of patients with cystic fibrosis (CF) (1–3). Coinfection with both pathogens, particularly P. aeruginosa and methicillin-resistant S. aureus (MRSA), has been associated with worsened patient outcomes (2, 4, 5). Both antagonistic and synergistic interactions have been observed between P. aeruginosa and S. aureus (2, 6). For example, P. aeruginosa can produce the virulence factor 2-heptyl-4-hydroxyquinoline *N*-oxide (HQNO), which inhibits S. aureus growth and drives S. aureus into a fermentative metabolic state (7, 8) but consequently decreases the susceptibility of S. aureus to multiple antimicrobials (9, 10). While S. aureus dominates CF lung infections early in life, P. aeruginosa tends to take over as patients age, often accompanied by worsening lung function (1, 3, 11). Continued understanding of interactions and population dynamics between these two pathogens is likely to result in improved therapeutic strategies and control of chronic infection in the CF lung.

Metabolomics profiling is increasingly being applied as a method to study polymicrobial communities (12–15) and to identify *in vivo* biomarkers of infection (16–19). Metabolomics profiling of sputum samples has enabled the identification of host-derived markers of CF (20); however, the high degree of metabolic and microbial heterogeneity across sputum samples makes it difficult to deconvolute the metabolic contributions of individual microbial species (20, 21). Alternatively, P. aeruginosa grown in the defined physiologically relevant media, synthetic cystic fibrosis media (SCFM), exhibits a transcriptional program similar to P. aeruginosa grown in CF sputum (22), providing a more reproducible way to study and compare the primary metabolism of individual species and strains. *In vitro* metabolomic profiling has enabled an improved understanding of P. aeruginosa metabolism as a function of altered media environments (23, 24), heterogeneous environmental backgrounds (25), variable antimicrobial resistance and virulence profiles (26), and coculture with S. aureus (15). While others have profiled the metabolome of P. aeruginosa (15, 23–27), to our knowledge, there are no open-source untargeted metabolomics data sets of multiple strains of both P. aeruginosa and S. aureus grown independently in a lung-like medium. Despite appreciated discrepancies between *in vitro* and *in vivo* metabolomics profiles (20), identification of robust cross-strain metabolic profiles for P. aeruginosa and S. aureus may help to guide the clinical study of pathogen-pathogen interactions and selection of candidate biomarkers of infection.

Here, we profiled the metabolism of multiple strains of P. aeruginosa and S. aureus grown in monoculture in SCFM (22). We show from untargeted metabolomics of culture supernatant that each species had a distinct metabolic signature despite strain-to-strain differences in significantly consumed or produced metabolites. We further compared metabolite consumption across species and found that P. aeruginosa and S. aureus robustly consume a shared subset of 15 nutrients. This result suggests that, even in the absence of P. aeruginosa-mediated growth inhibition and killing, S. aureus may be required to compete with P. aeruginosa for key nutrients in coculture. This result is consistent with the previous observation that coexistence between P. aeruginosa and S. aureus in brain heart infusion (BHI) was associated with a transcriptional shift away from glucose metabolism in S. aureus (28). Finally, we identify metabolites uniquely produced by each species across all tested strains. Together, our findings provide new insight into the capacity for competitive metabolic interaction between P. aeruginosa and S. aureus and suggest species-specific biomarkers of infection with the potential to improve treatment and diagnosis of CF lung infections.

## RESULTS

### Untargeted metabolomics of P. aeruginosa and S. aureus in monoculture in a lung-like media.

**FIG 1:**
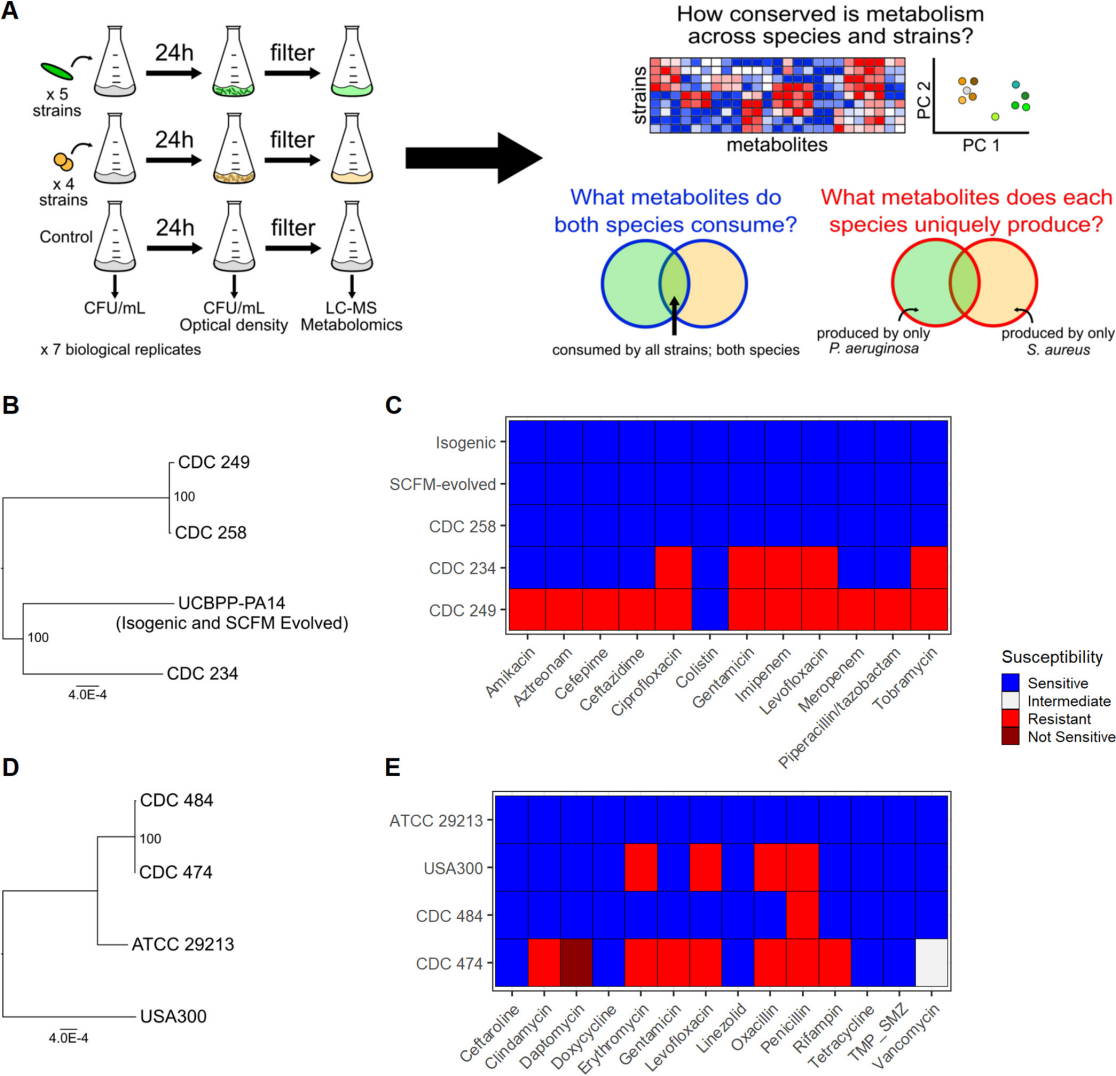
Summary of experimental design and bacterial strains used in this study. (A) Five strains of P. aeruginosa (green) and four strains of S. aureus (yellow) were each grown individually in SCFM for 24 h. Cultures were filter sterilized, and LC-MS was performed on supernatants. SCFM medium was also profiled as a control. Strain-specific metabolic profiles, cross-species metabolite consumption, and species-specific metabolite production were identified. (B and D) Phylogenetic trees of P. aeruginosa (B) and S. aureus (D) strains. Reference genomes were used for laboratory strains UCBPP-PA14, ATCC 29213, and USA300. A single reference genome was used for isogenic and SCFM-evolved UCBPP-PA14 given their shared history and highly similar metabolic functionality. (C and E) Antimicrobial susceptibility profiles of P. aeruginosa (C) and S. aureus (E) strains.

Five strains of P. aeruginosa and four strains of S. aureus were grown individually in monoculture in SCFM (Fig. 1A). The strains came from various sources (e.g., laboratory, clinical) and genetic backgrounds and displayed various levels of antimicrobial susceptibility and pigment production (Table 1; Fig. 1B to E; Fig. S1 in the supplemental material). P. aeruginosa strain UCBPP-PA14 was profiled both before (isogenic) and after (SCFM evolved) 10 days of laboratory adaptation to SCFM (Fig. S2; see Materials and Methods). This was done to evaluate the sensitivity of P. aeruginosa metabolism to heterogeneous recent environmental backgrounds. Additionally, the S. aureus laboratory strains ATCC 29213 (methicillin sensitive) and USA300 LAC (methicillin resistant) and five isolates from the CDC and FDA Antibiotic Resistance Isolate Bank (29; https://www.cdc.gov/drugresistance/resistance-bank/index.html) were selected (Table 1). Interestingly, strains with highly similar genomes could display dissimilar antimicrobial susceptibility profiles (Fig. 1B to E). Untargeted metabolomics data were generated from the supernatant of each strain and compared to unconditioned media to evaluate strain- and species-specific metabolite consumption and production.

**TABLE 1:** Laboratory strains and clinical isolates used in this study

Identifier	Species	Strain	Sequence (accession no.)	Reference or source
Isogenic	P. aeruginosa	UCBPP-PA14	GenBank accession no. NC_008463.1	47, 57
SCFM evolved	P. aeruginosa	Adapted UCBPP-PA14	GenBank accession no. NC_008463.1	This study (Materials and Methods), 57^*a*^
CDC258	P. aeruginosa	Clinical	SRA accession no. SRR4417541	29
CDC234	P. aeruginosa	Clinical	SRA accession no. SRR4417559	29
CDC249	P. aeruginosa	Clinical	SRA accession no. SRR5122332	29
ATCC 29213	S. aureus	ATCC 29213	Assembly accession no. GCF_001267715.1	58, 59^*a*^
USA300	S. aureus	USA300 LAC	GenBank accession no. NC_007793.1	This study (Acknowledgments), 60^*a*^
CDC484	S. aureus	Clinical	SRA accession no. SRR8526670	29
CDC474	S. aureus	Clinical	SRA accession no. SRR6985717	29

a Source of sequence data if different from source of isolate (e.g., reference genome).

10.1128/mSystems.00480-21.1 FIG S1 Representative image of spent SCFM of P. aeruginosa strains. From left to right, tubes contain filtered supernatant from cultures of isogenic P. aeruginosa, SCFM-evolved P. aeruginosa, CDC258, CDC234, and CDC249. Download FIG S1, JPG file, 0.2 MB. Copyright © 2021 Dunphy et al. 2021 Dunphy et al. https://creativecommons.org/licenses/by/4.0/ This content is distributed under the terms of the Creative Commons Attribution 4.0 International license.

10.1128/mSystems.00480-21.2 FIG S2 Growth curves and dynamics of SCFM-evolved P. aeruginosa during and postadaptation. (A) Daily growth curves of P. aeruginosa while being adapted to SCFM. (B) Growth rate of the SCFM-evolved strain on each day of the adaptation. (C) Median growth curves in SCFM of SCFM-evolved and isogenic P. aeruginosa postadaptation across five biological replicates. Shaded region denotes interquartile range. OD_600_ is background subtracted. Download FIG S2, JPG file, 0.3 MB. Copyright © 2021 Dunphy et al. 2021 Dunphy et al. https://creativecommons.org/licenses/by/4.0/ This content is distributed under the terms of the Creative Commons Attribution 4.0 International license.

**FIG 2:**
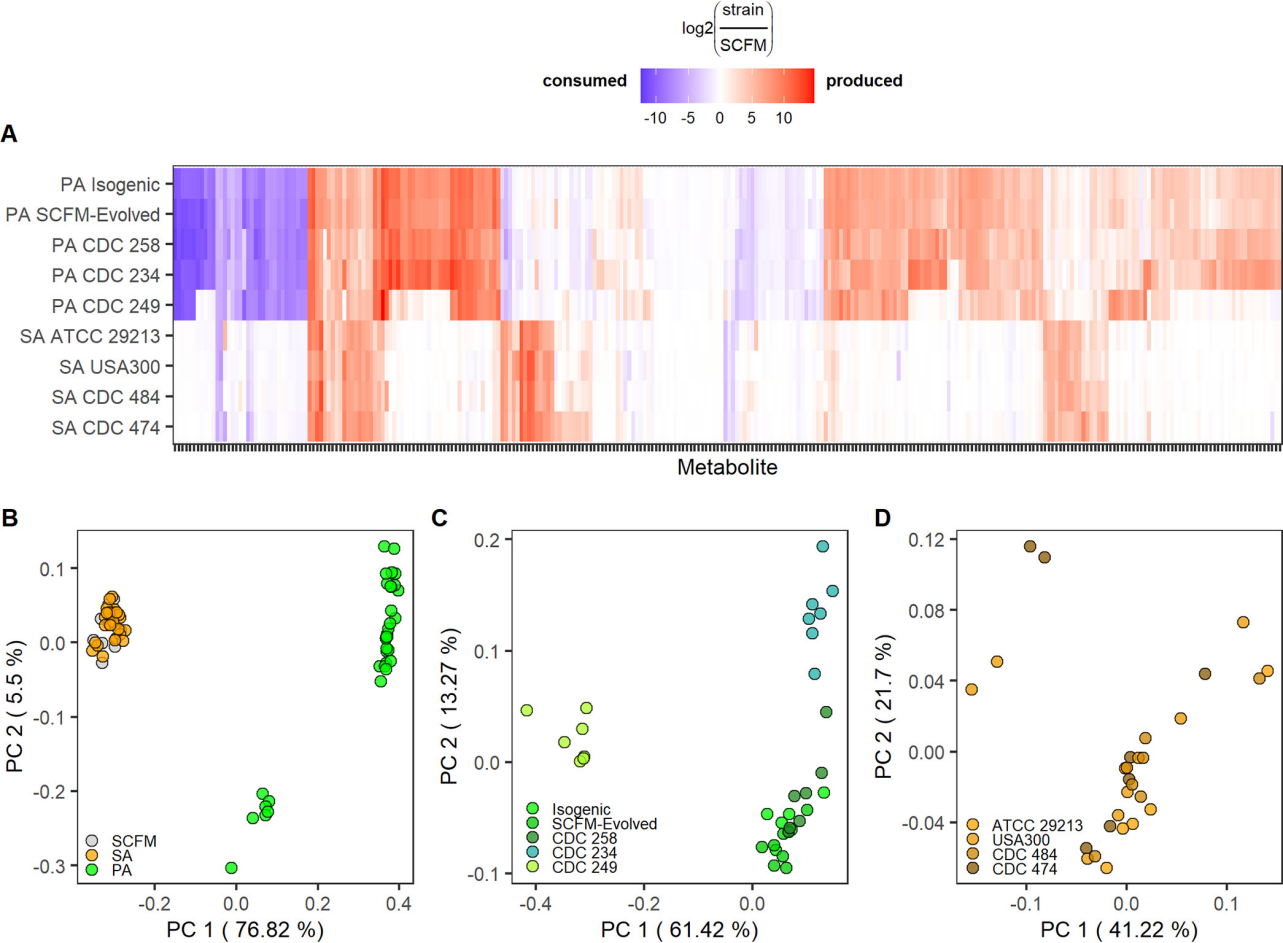
Metabolic profiles of P. aeruginosa and S. aureus grown in monoculture in SCFM. (A) Metabolomics profiles of conditioned SCFM. Metabolites significantly altered relative to SCFM in at least one condition are shown (*P* < 0.05). Metabolites clustered by Euclidean distance with complete linkage. Significance determined by Wilcoxon rank sum test with Benjamini-Hochberg correction. (B to D) PCoA of Bray-Curtis distances calculated using all raw peak areas for all replicates of all strains (B), raw peak areas for P. aeruginosa strains only (C), or S. aureus strains only (D). Percent variance is shown for each principal coordinate. *n* = 7 replicates for all conditions.

Out of 314 metabolites identified by untargeted metabolomics, 288 were significantly altered (*P* < 0.05) in the supernatant of at least one strain relative to unconditioned media. P. aeruginosa strains exhibited generally higher log_2_ fold change (log_2_FC) than S. aureus strains in significantly consumed or produced metabolites (Fig. 2A). Additionally, clustering the raw peak areas of all 314 identified metabolites with principal coordinate analysis (PCoA) revealed that P. aeruginosa strains were distinct from S. aureus profiles, which were relatively similar to unconditioned SCFM controls (Fig. 2B). Both of these results corresponded with the observation that P. aeruginosa strains grew to higher culture densities than S. aureus strains in SCFM (Fig. S3). Individual clustering of each species further highlighted strain-specific differences in P. aeruginosa metabolomes (Fig. 2C), while no clear strain-specific profiles emerged in S. aureus metabolomes (Fig. 2D). These results demonstrate the ability of each pathogen to alter the metabolic landscape of a lung-like environment.

10.1128/mSystems.00480-21.3 FIG S3 Growth of P. aeruginosa and S. aureus prior to metabolomics sampling. (A) CFU/ml at the time of inoculation (*t* = 0 h) and collection for metabolomics (*t* = 24 h). CFU/ml are reported as log_10_(CFU/ml + 1). (B) OD_600_ for all replicates and strains at 24 h. Black denotes median, and error bars denote interquartile range across all seven biological replicates. Download FIG S3, JPG file, 0.4 MB. Copyright © 2021 Dunphy et al. 2021 Dunphy et al. https://creativecommons.org/licenses/by/4.0/ This content is distributed under the terms of the Creative Commons Attribution 4.0 International license.

### Metabolite production and consumption were mostly conserved across strains of the same species.

**FIG 3:**
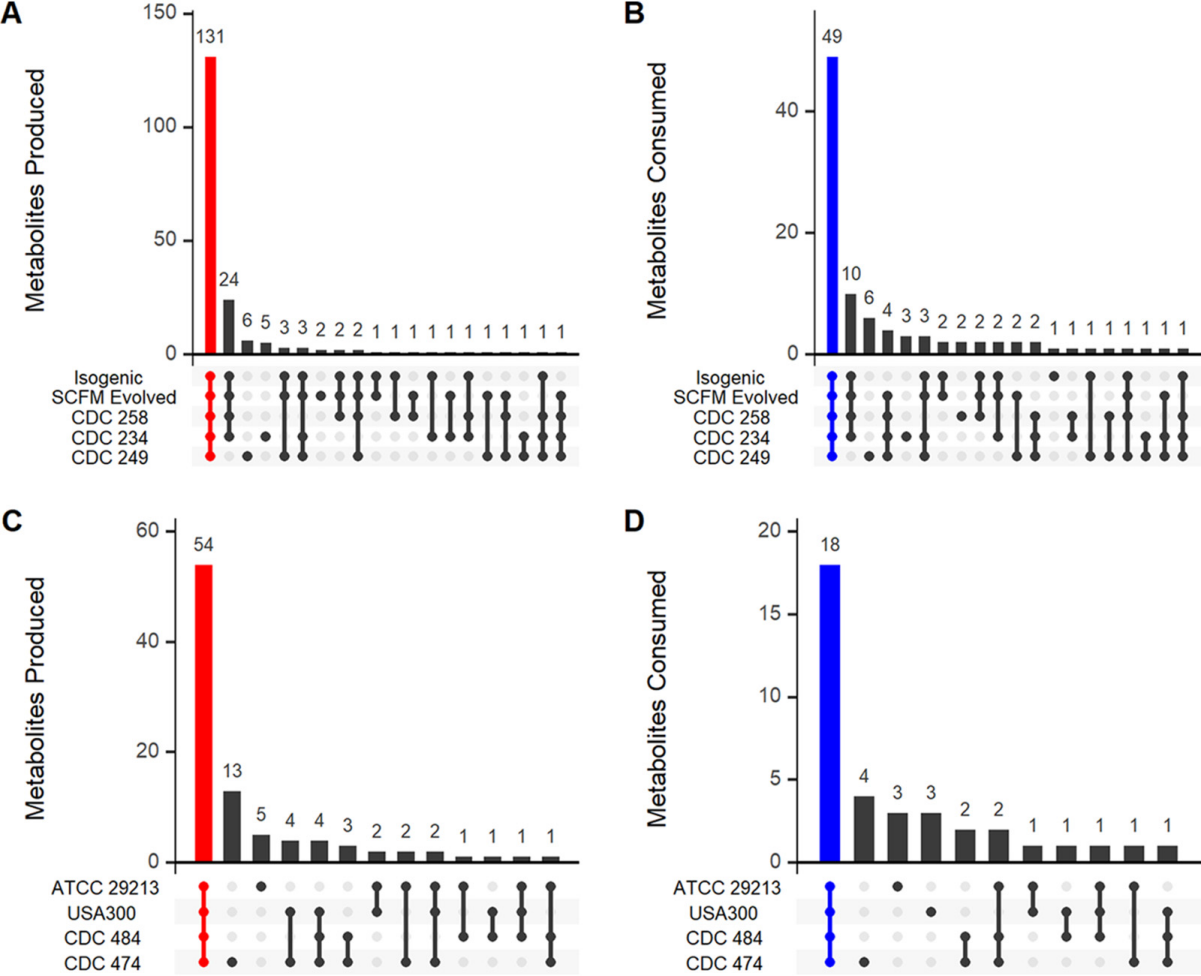
Intraspecies strain-specific metabolite production and consumption. Number of significant metabolites produced (*P* < 0.05, log_2_FC > 0) or consumed (*P* < 0.05, log_2_FC < 0) by subsets of P. aeruginosa strains (A and B) or S. aureus strains (C and D). Metabolites produced by all strains of a species are shown in red. Metabolites consumed by all strains of a species are shown in blue.

Overlap between intraspecies extracellular metabolomes was evaluated to identify robust species-specific metabolic signatures and assess strain-to-strain metabolic variability (Fig. 3). For the five strains of P. aeruginosa, the majority of metabolites produced (131 metabolites, *P* < 0.05, log_2_FC > 0) and consumed (49 metabolites, *P* < 0.05, log_2_FC < 0) were conserved (Fig. 3A and B). Metabolism was highly similar between the isogenic and SCFM-evolved strains (235/256 metabolites, *P* < 0.05), indicating that prior adaptation to SCFM did not have a strong impact on metabolite consumption or production.

Similar to what was seen in P. aeruginosa, the majority of metabolites produced by S. aureus (54 metabolites, *P* < 0.05, log_2_FC > 0) were produced by all four strains (Fig. 3C). S. aureus strains reached lower final growth densities than P. aeruginosa strains (Fig. S3), and metabolite consumption by S. aureus was more variable, with 18 conserved metabolites and 19 metabolites only consumed by subsets of the four strains (Fig. 3D). Overall, we observed a high degree of consistency across metabolites significantly consumed and produced by strains of the same species, highlighting a shared core metabolic program in a CF lung-like environment. Intraspecies differences in metabolism additionally suggest the potential for less robust, strain-specific metabolic interactions between P. aeruginosa and S. aureus.

### A subset of metabolites was robustly consumed by both P. aeruginosa and S. aureus.

**FIG 4:**
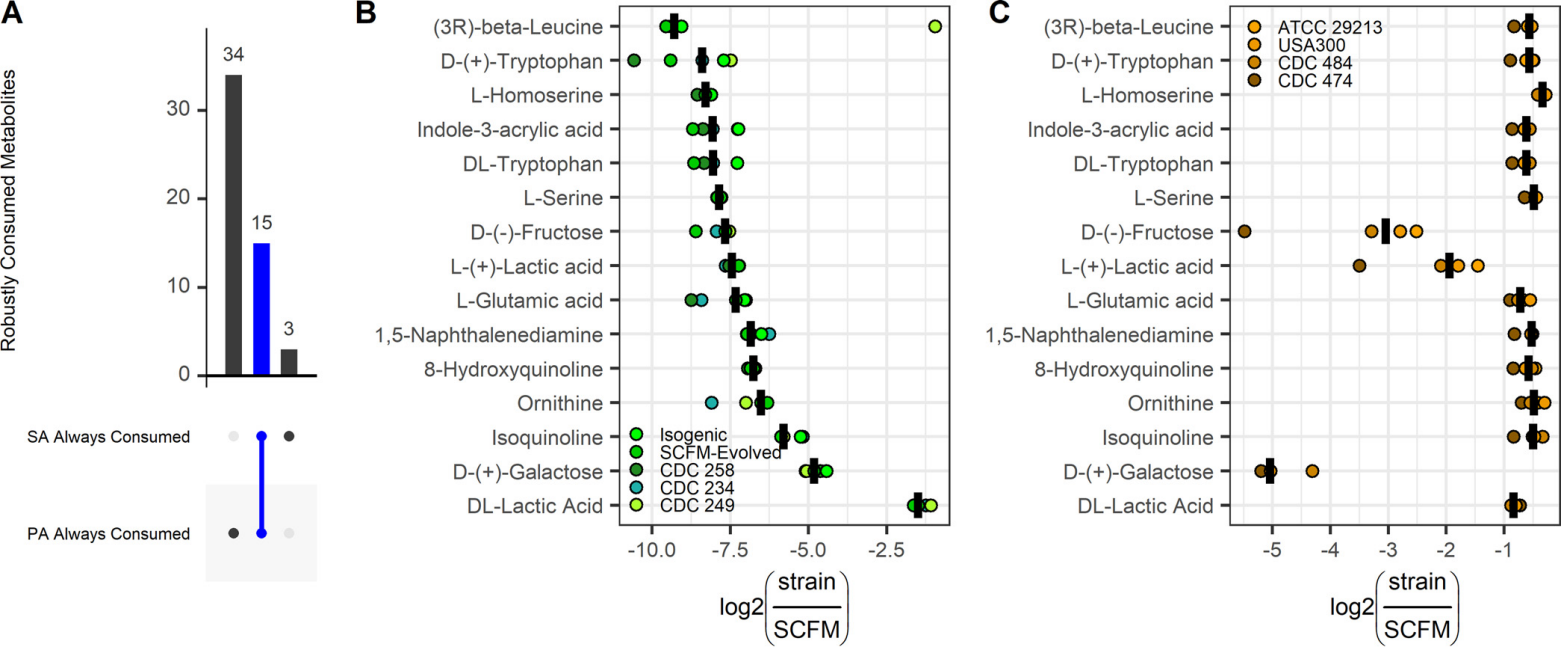
Shared metabolite consumption between P. aeruginosa and S. aureus in a lung-like medium. (A) Number of significant metabolites robustly consumed across all tested monocultures of P. aeruginosa and S. aureus (*P* < 0.05, log_2_FC < 0). Log_2_FCs in metabolites consumed by both P. aeruginosa (B) and S. aureus (C) relative to SCFM. Crossbar denotes median change across strains.

In order to predict nutrients that P. aeruginosa and S. aureus may compete over when colocalized in the lung or *in vitro*, metabolite consumption was compared across all strains of each species. Metabolomics data revealed 15 metabolites significantly consumed (*P* < 0.05, log_2_FC < 0) by all nine profiled strains (Fig. 4A), indicating that the majority of metabolites robustly consumed by S. aureus were also robustly consumed by P. aeruginosa. These metabolites included several key amino acids (e.g., tryptophan, l-serine, l-glutamic acid, and ornithine) and lactic acid (Fig. 4B and C). Additionally, all nine strains also consumed glucose from the media (Fig. S4). These results suggest that in addition to direct suppression of S. aureus growth by P. aeruginosa (6, 8, 30), P. aeruginosa and S. aureus have the potential to metabolically compete over key nutrients when cocultured in a lung-like media.

10.1128/mSystems.00480-21.4 FIG S4 Relative glucose consumption in conditioned SCFM. Black points denote medians, and error bars denote interquartile ranges across all seven biological replicates of each strain. Download FIG S4, JPG file, 0.2 MB. Copyright © 2021 Dunphy et al. 2021 Dunphy et al. https://creativecommons.org/licenses/by/4.0/ This content is distributed under the terms of the Creative Commons Attribution 4.0 International license.

### Robust species-specific-produced metabolites are potential biomarkers of lung infection.

**FIG 5:**
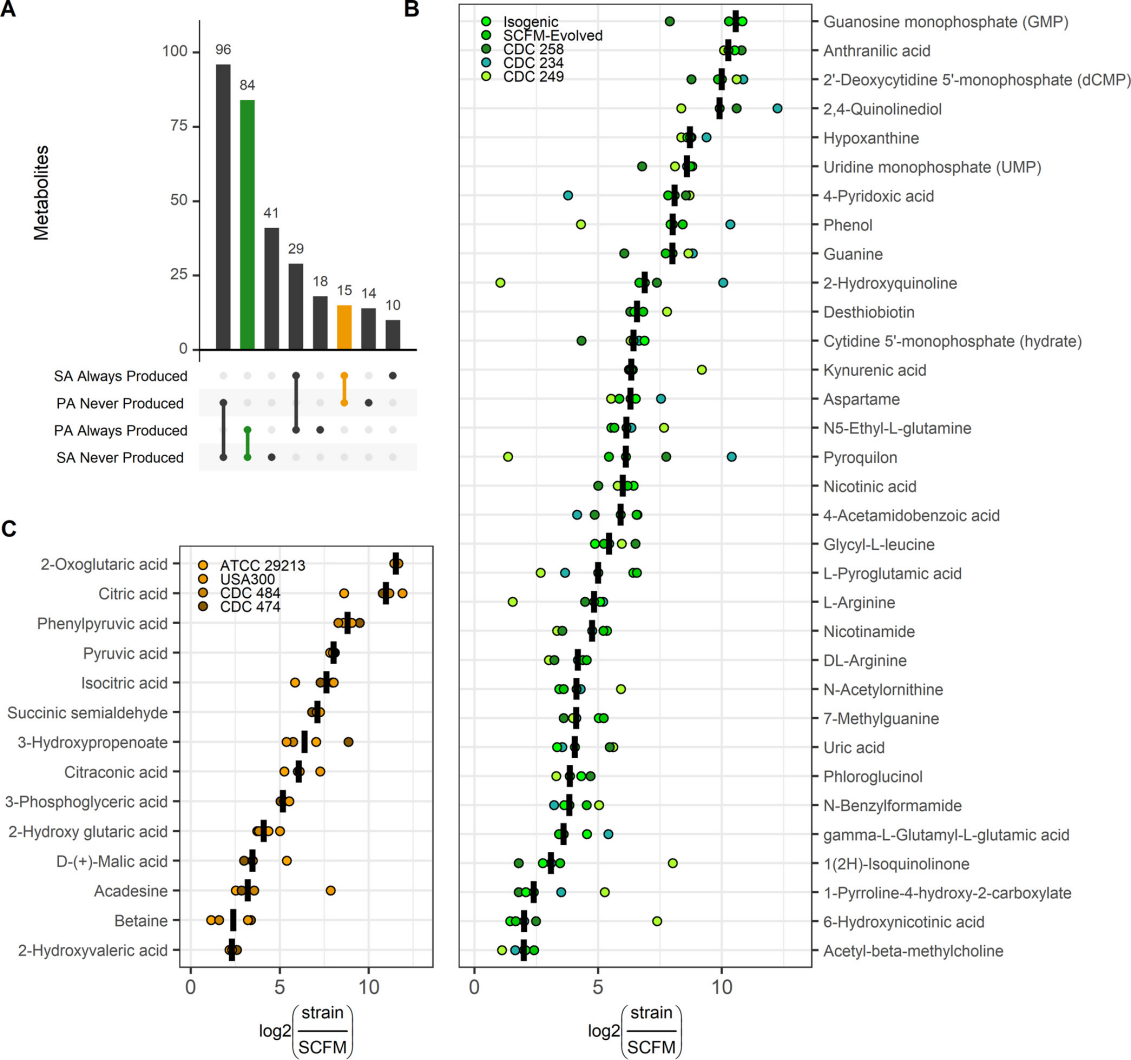
P. aeruginosa and S. aureus specific metabolite production in a lung-like medium. (A) Number of metabolites that were always or never significantly produced across strains of each species (*P* < 0.05, log_2_FC > 0). Metabolites uniquely produced by P. aeruginosa strains are shown in green. Metabolites uniquely produced by S. aureus strains are shown in yellow. Log_2_FCs in metabolites produced by only P. aeruginosa (B) or S. aureus (C) strains. Only metabolites with a minimum log_2_FC of ≥1 across all strains within a species are shown. Metabolites produced by P. aeruginosa without an associated KEGG ID were excluded. Crossbar denotes median change of each metabolite across strains.

Metabolites that are produced uniquely by a single species have the potential to be developed into biomarkers of P. aeruginosa or S. aureus infection. In total, there were 84 metabolites that were statistically significantly (*P* < 0.05, log_2_FC > 0) produced by all five strains of P. aeruginosa and not produced by S. aureus. Conversely, there were 15 metabolites produced uniquely by all four strains of S. aureus (Fig. 5A). In an effort to account for potential noise of the environmental milieu, only significantly altered metabolites with a minimum log_2_FC of >1 across all strains of each species are shown (Fig. 5B and C; Data Set S1).

10.1128/mSystems.00480-21.5 DATA SET S1 Fold changes, log_2_FCs, unadjusted *P* values, and adjusted *P* values for all measured metabolites. Download Data Set S1, CSV file, 0.3 MB. Copyright © 2021 Dunphy et al. 2021 Dunphy et al. https://creativecommons.org/licenses/by/4.0/ This content is distributed under the terms of the Creative Commons Attribution 4.0 International license.

Metabolites produced uniquely by P. aeruginosa relative to unconditioned SCFM included nucleic acids and amino acids, their derivatives, as well as a downstream quinoline from the Pseudomonas quinolone signal (PQS) system (31) (Fig. 5B). Anthranilic acid and kynurenic acid, known downstream products of tryptophan metabolism (32), were both highly produced (log_2_FC > 5) in P. aeruginosa-conditioned SCFM supernatants. Downstream metabolites of several amino acids, including nicotinamide, nicotinic acid, and the P. aeruginosa-specific metabolite 6-hydroxynicotinc acid (33), were also increased. Although these metabolites were not seen in S. aureus-conditioned SCFM supernatants, more research is required to determine whether they are unique to P. aeruginosa and can be distinguished from the host milieu. Interestingly, the metabolite, 2,4-quinolinediol (DHQ), which has been shown to increase virulence and maintain pyocyanin production in model organisms (31), was highly elevated in supernatants of all five tested strains of P. aeruginosa. DHQ has been measured in CF sputum samples (31), suggesting that it has the potential to be a strong candidate biomarker for P. aeruginosa lung infection.

Although S. aureus did not grow as robustly in SCFM as P. aeruginosa, all four strains produced a shared set of 54 metabolites (Fig. 3C), 15 of which were not significantly produced by any strains of P. aeruginosa (Fig. 5A). S. aureus produced fewer unique metabolites than P. aeruginosa, and these metabolites consisted mostly of glycolysis or tricarboxylic acid (TCA) cycle components and their derivatives (Fig. 5C). The metabolite with the largest fold change from unconditioned media, 2-oxoglutaric acid, is known to be produced during catabolism of l-glutamic acid and other amino acids (34). We hypothesize that these components were secreted following amino acid degradation and not consumed because other preferred carbon sources were still freely available (35). Consequently, it is unclear whether these metabolites would remain available in the host milieu or whether they would be taken up by S. aureus in the absence of glucose or by other microbes or the host. For example, it has been previously shown that P. aeruginosa is capable of catabolizing citric acid (36). We conclude that while S. aureus produces metabolites that P. aeruginosa does not, known S. aureus-specific proteins (e.g., hemolysins) may be more robust and unique biomarkers of S. aureus infection in sputum. It is worth noting, however, that S. aureus has been shown to produce fewer virulence factors *in vitro* than in CF sputum (37).

## DISCUSSION

Here, we have shown that P. aeruginosa and S. aureus have distinct metabolomes, with the majority of significantly altered metabolites conserved within strains of each species (Fig. 3). We further identified a subset of 15 metabolites that were significantly consumed by both species, including the known sputum components l-lactic acid, l-glutamic acid, l-serine, DL-tryptophan, and ornithine (Fig. 4) (22). Glucose was additionally consumed by all strains (Fig. S4). These robustly consumed metabolites suggest a baseline level of nutrient competition between P. aeruginosa and S. aureus in a CF lung-like environment. Finally, metabolites are increasingly being considered biomarkers of infection (16–19), and here, we proposed a subset of produced metabolites involved in l-tryptophan catabolism and PQS signaling as potential unique biomarkers of P. aeruginosa infection in the CF lung (Fig. 5B).

Although P. aeruginosa and S. aureus are known to coinfect the lung, *in vitro* cocultures of these two organisms can be difficult to maintain (8, 38). In addition to known mechanisms of hostility of P. aeruginosa toward S. aureus to directly kill or drive S. aureus into a less metabolically active state (2), we propose that P. aeruginosa and S. aureus coculture may be further complicated by the versatile nature of P. aeruginosa catabolism. In addition to the 15 metabolites consumed by all strains of both species, P. aeruginosa robustly consumed another 34 metabolites (Fig. 4), indicating that, barring any condition-specific or coculture-driven shifts in carbon source catabolism (39), there may be few nutrients uniquely available to S. aureus in coculture. Given the large overlap in metabolite consumption, we hypothesize that it may be unlikely that P. aeruginosa and non-small colony variants of S. aureus stably colocalize closely enough in the CF lung to impact one another’s nutrient environments. That said, adaptation to ciprofloxacin exposure has been shown to result in a loss of catabolic function in P. aeruginosa (36), and therefore, treatment-driven catabolic deficits may lessen the potential for nutrient competition and promote coexistence of the two pathogens *in vivo*.

P. aeruginosa-conditioned SCFM contained molecules known to be involved in PQS signaling and virulence. Specifically, anthranilic acid, a precursor of quinoline signaling (40, 41), and the quinoline DHQ (31, 42) were consistently detected in the supernatant of all five strains (Fig. 5B). Anthranilic acid is an important precursor and suggested therapeutic target for PQS signaling and can be derived from l-tryptophan catabolism or chorismate (32, 43, 44). Consumption of l-tryptophan (Fig. 4B) and production of kynurenic acid (Fig. 5B), another product of l-tryptophan catabolism (32), suggest that l-tryptophan was the main precursor of anthranilic acid in the conditioned SCFM, consistent with literature that this is the main source of anthranilic acid in rich media (43). DHQ is a nonalkylated quinoline that is capable of being produced in both aerobic and anaerobic environments and, in a previous study, was detected in 34/80 sputum samples taken from 45 cystic fibrosis patients with histories of cultures positive for P. aeruginosa (31). Additionally, the well-studied quinoline, HQNO, was significantly produced in all P. aeruginosa strains with the exception of CDC249 (Data Set S1 in the supplemental material) and has been previously found in sputum of CF patients with P. aeruginosa infections (20). Taken together, given that l-tryptophan is reliably found in CF sputum (22), PQS precursors and additional quinolines derived from l-tryptophan catabolism have the potential to be robust biomarkers of P. aeruginosa infection in the CF lung.

Although we identified species-specific metabolites that were produced across multiple strains and that were detected by others in CF sputum (20, 32), there are three limitations of our study that may impact the robustness of our proposed biomarkers of P. aeruginosa infection. First, the sources (e.g., lung, urine, etc.) of clinical isolates used in this study are unknown, and therefore, it is unclear how similar they are to isolates adapted to a CF lung environment. Second, it is possible that metabolite production may be sensitive to *in vivo* stressors (e.g., host-pathogen interactions) that were not captured by our experimental design. For example, while absent from SCFM, mucins are highly abundant in the CF lung (45) and have been shown to impact the expression of genes involved in virulence factor production in P. aeruginosa (46). Finally, although our proposed biomarkers were produced across five diverse strains of P. aeruginosa, a single metabolite may not be sufficient to reliably detect P. aeruginosa infection. A stronger diagnostic could likely be developed with a signature of multiple metabolites identified in our study and from previous literature. Future studies should focus on measuring identified biomarkers in a large number of sputum samples from cystic fibrosis patients with and without P. aeruginosa lung infections to assess marker relevance.

In summary, we have found that P. aeruginosa and S. aureus produce distinct metabolomic profiles when grown in SCFM with limited strain-to-strain variability in significantly altered metabolites, indicating that while many metabolic interactions between the two organisms may be conserved, others may be strain-specific. Characterization of robust species-specific metabolism and intraspecies metabolic variability in physiologically relevant environments is important for the study of polymicrobial infections, especially in cases where organisms are challenging to grow in coculture *in vitro*. Furthermore, high-quality metabolomics data sets in well-established defined media are useful for interrogating cross-species and cross-strain questions of interest. A better understanding of polymicrobial interactions and more rapid and reliable species detection in sputum have the potential to greatly improve treatment of CF lung infections.

## MATERIALS AND METHODS

### Bacterial strains and media conditions.

Three lab strains, one laboratory-adapted lab strain, and five clinical isolates were selected for metabolomics profiling (Table 1). P. aeruginosa strain UCBPP-PA14 (47), methicillin-sensitive S. aureus strain ATCC 29213, and methicillin-resistant S. aureus strain USA300 LAC were selected as representative laboratory strains. P. aeruginosa strain UCBPP-PA14 was additionally adapted to SCFM for 10 days as described below. Clinical isolates were provided by the CDC and FDA Antibiotic Resistance Isolate Bank (29; https://www.cdc.gov/drugresistance/resistance-bank/index.html). Specifically, three clinical isolates from the “Pseudomonas aeruginosa” panel (ID 234, ID 249, and ID 258) and two clinical isolates from the “Staphylococcus with Borderline Oxacillin Susceptibility” panel (ID 474 and ID 484) were selected due to their differing levels of antibiotic susceptibility.

For supernatant collection, cultures were revived from frozen on lysogeny broth (LB) agar plates, inoculated into LB liquid media, and washed in 1× Dulbecco's phosphate-buffered saline (DPBS) prior to inoculation into SCFM. SCFM was prepared and stored at 4°C as previously described (22). Stocks of SCFM components were remade as they were depleted or if precipitation or a change in color was observed. SCFM was selected over SCFM2 (48) to maximize the number of media components that could be detected in our metabolomics analysis as well as to avoid challenges associated with viscous samples (49).

### Generation of an SCFM-evolved P. aeruginosa strain.

P. aeruginosa strain UCBPP-PA14 (isogenic) was evolved to SCFM for 10 days prior to metabolomics profiling to create an SCFM-evolved P. aeruginosa isolate. Briefly, isogenic P. aeruginosa was streaked onto an LB agar plate and incubated for 24 h at 37°C. A single colony was inoculated into 5 ml of SCFM, and 200 μl of this culture was moved to a single well in a 96-well plate. Throughout the adaptive evolution experiment, the SCFM-evolved lineage was incubated at 37°C with shaking, and the optical density at 600 nm (OD_600_) of the lineage was measured every 10 min (Tecan Infinite M200 Pro; Fig. S2A in the supplemental material). Every 24 h, 5 μl of culture was propagated into 195 μl of fresh SCFM, resulting in a 1:40 dilution. The remaining sample was frozen at a final concentration of 25% glycerol. This process was repeated for 10 days when growth rates had leveled out for 5 days (Fig. S2B) with the exception of between day 5 and day 6, where the lineage was revived from frozen to inspect and confirm homogeneity.

As a final check to ensure homogeneity, the day 10 SCFM-evolved P. aeruginosa was streaked onto an LB plate and incubated for 22 h at 37°C. A single colony was selected and inoculated into 5 ml of SCFM. This culture was grown for 26 h with shaking at 200 rpm and 37°C. Liquid culture from this sample was frozen at a final concentration of 25% glycerol, and this frozen stock was used as the SCFM-evolved lineage. The growth curves of the SCFM-evolved lineage and isogenic P. aeruginosa in SCFM were measured (Fig. S2C). Growth rates were calculated with the growthrates package in R (50).

### Sequence analysis.

Whole genomes or references of all isolates except for the SCFM-evolved P. aeruginosa strain were publicly available (Table 1). All genomic analyses were performed through the Pathosystems Resource Integration Center (PATRIC) (51). Raw reads of clinical isolates were assembled using the auto assembly strategy with default parameters. Assemblies of CDC isolates and ATCC 29213 were annotated using default settings. Reference genomes for UCBPP-PA14 and USA300_FPR3757 were identified within PATRIC. Finally, phylogenetic trees of P. aeruginosa and S. aureus strains were generated using the codon tree method. Default parameters were used with the exception of the number of genes included in the analysis, which was increased to 1,000 to ensure resolution of closely related genomes.

### Susceptibility profiles of bacterial strains.

Susceptibility profiles of clinical isolates from the CDC and FDA Antibiotic Resistance Isolate Bank were reported as found on their database (https://www.cdc.gov/drugresistance/resistance-bank/index.html). Susceptibility profiling was performed on the remaining laboratory strains (isogenic P. aeruginosa, SCFM-evolved P. aeruginosa, ATCC 29213, and USA300) by the Clinical Microbiology Laboratory at the University of Virginia (UVA) Health System. The susceptibility of lab strains to all antibiotics was measured by broth microdilution with the exception of penicillin, which was measured using a Kirby-Bauer disk diffusion assay paired with a beta-lactamase test. Specifically, S. aureus laboratory strains were profiled with Vitek 2 Systems (bioMérieux Inc.), and P. aeruginosa laboratory strains were profiled with a Sensititre Aris 2× (Thermo Scientific). All susceptibility profiling at UVA was performed in accordance with the Clinical and Laboratory Standards Institute (CLSI), and breakpoints were consistent with the 29th edition of the CLSI M100 (52).

### Collection of conditioned SCFM.

Isolates were streaked from frozen stocks onto LB agar plates and incubated overnight at 37°C. Single colonies from each plate were inoculated into 5 ml of LB liquid media in 50-ml flasks and incubated at 37°C with shaking (200 rpm) overnight. Liquid cultures were washed twice in 1× DPBS and a third time in SCFM (6,000 rpm for 5 min). Washed cultures were inoculated into 25 ml of SCFM in 500-ml flasks at an OD_600_ of 0.01 (∼10^4^ to 10^7^ CFU/ml) and were incubated at 37°C with shaking (200 rpm) for 24 h. As a control, a flask of SCFM with no bacteria was also incubated under the same conditions. Stationary cultures were centrifuged (3,000 rpm at 4°C for 20 min), and supernatants were collected, filter sterilized (22-μm PES membrane), and stored at 4°C. Colony counts were measured at the time of inoculation and just prior to collection of supernatant and reported as CFU per milliliter (Fig. S3A). The OD_600_ of each washed culture was also measured at the time of supernatant collection (Fig. S3B). Seven biological replicates were each collected on a different day, where each replicate started from an individual colony. Four batches of SCFM were used across the seven replicates.

### Metabolite extraction and tandem mass spectrometry analysis of the metabolites.

Supernatants were delivered fresh without freezing to the UVA Biomolecular Analysis Facility and stored at −80°C upon receipt until preparation. Approximately 150 μl of supernatants were mixed with 600 μl of ice-cold methanol and vortexed for 30 s. Samples were then centrifuged at maximum speed for 25 min at 4°C. For each sample, 600 μl (650 μl for a pooled sample) was dried in a SpeedVac and stored at −80°C until use. Prior to liquid chromatography-mass spectrometry (LC-MS) measurement, samples were reconstituted with 100 μl 0.1% formic acid and 5% methanol in water, centrifuged for 10 min, and transferred to LC vials (80 μl/sample).

Samples were analyzed in untargeted fashion by liquid chromatography-high resolution mass spectrometry (LC-HRMS). Samples were injected in randomized fashion via a Thermo Vanquish UHPLC, and separation of the metabolites was achieved using Thermo Accucore C_18_ column (Thermo Scientific; 2.1 by 100 mm, 1.5 μm) maintained at 50°C. The injection volume was 2 μl. For the 15-minute gradient, mobile phase consists of solvent A (0.1% formic acid in water) and solvent B (0.1% formic acid in methanol). The gradient was as follows: 0 to 8.0 min 50% solvent B, 8.0 to 13.0 min held at 98% solvent B, and 13.1 to 15.0 min revert to 0% solvent B to reequilibration for next injection. Spectra were acquired on Thermo ID-X Tribrid MS, using both positive and negative mode. Data were acquired in full MS mode (1 μscan) at a resolution of 120,000 with a scan range of 67 to 1,000 *m/z* at a normalized automatic gain control (AGC) target of 25%, and 50 ms of maximum injection time was allowed. RF lens amplitude was set at 35%. A heated electrospray ionization (HESI) source was operated at 3.5 kV and 2.5 kV for positive and negative modes, respectively. Ion source sheath gas was set at 35 and auxiliary gas at 7. Ion transfer tube temperature was maintained at 275°C, while vaporizer temperature was maintained at 320°C. Tandem MS was performed by applying quadrupole isolation with an isolation window of 1.6 *m/z*. Activation type was set at high-energy collisional dissociation (HCD), and masses were fragmented with HCD assisted collision energy (%) of 153,550. Fragment masses were detected by Orbitrap at a resolution of 30,000.

Raw spectra were analyzed in Compound Discoverer 3.1 with standard settings. For precursor selection, MS(n-1) precursors were selected, and signal-to-noise (S/N) threshold was set at 1.5. Retention time alignment was performed using the adaptive curve algorithm with maximum shift allowed for 2 min at a mass tolerance of 5 ppm. Compounds were detected at a mass tolerance of 5 ppm, and minimum peak intensity threshold was set at 500,000. Preferred ions were set at either [M+H] or [M−H], respectively, for positive or negative mode. Compounds were grouped at a mass tolerance of 5 ppm, and RT tolerance of 0.2 min was allowed. A quality control (QC)-based area correction was applied using a linear regression model with minimum allowable QC coverage of 50%, and maximum QC area relative standard deviation (RSD) allowed was 30%. Peak areas were normalized using constant median. Blank samples were used for detection and identification of background peaks. Compound annotations were performed by searching the ddMS^2^ masses in mzCloud. A second search with either formula or exact mass was performed in ChemSpider, KEGG database, and an in-house database of IROA Mass Spectrometry Metabolite Library of Standards (MSMLS) (https://iroatech.com/). The in-house database contained approximate standards for 550 compounds detected in both positive and negative modes detected on the same Orbitrap mass spectrometer, using similar chromatographic separation. Metabolites were retained and considered sufficiently annotated if they met the following two conditions. First, metabolites were required to have an mzCloud or mzVault score of ≥60. Second, metabolites were required to have been verified during the second search. If a metabolite was detected in both positive and negative modes, the positive mode was kept, and the negative mode was discarded.

### Quantification of glucose consumption.

Glucose levels were determined using the MyQubit Amplex Red glucose assay as directed by the manufacturer (Thermo Fisher Scientific; catalog nos. A22189 and A33855; Fig. S4). Glucose measurements below the limit of detection were omitted. Glucose levels of each conditioned media sample were reported relative to unconditioned SCFM.

### Metabolomics data normalization and statistical analyses.

Raw peak areas for all samples and replicates were log_2_ transformed and mean-centered for each high-quality metabolite. Metabolites significantly altered in conditioned SCFM relative to unconditioned SCFM were identified by performing Wilcoxon rank-sum tests on log_2_-transformed, mean-centered data. The Benjamini-Hochberg method was used to correct for multiple hypothesis testing (53). Corrected *P* values of <0.05 were considered significant.

Log_2_FCs between conditioned media and unconditioned SCFM were calculated to visualize the direction of change of significantly altered metabolites (Fig. 2A; Fig. 4B and C; Fig. 5B and C). The log_2_FC was calculated as

$${\text{log}}_{\text{2}}[\frac{\text{median}({\text{RPA}}_{\text{strain}})}{\text{median}({\text{RPA}}_{\text{SCFM}})}]$$

for each metabolite, where RPA is the raw peak area, and the medians were taken across biological replicates for each condition. Log_2_FCs were used to define consumption (log_2_FC < 0) and production (log_2_FC > 0) of significantly altered metabolites.

### Multivariate analysis, clustering, and cross-group comparisons of metabolomics data.

Metabolite log_2_FCs were clustered hierarchically by Euclidean distance with complete linkage (Fig. 2A). PCoA was performed on Bray-Curtis distances between raw metabolomics profiles of all samples with the ape package in R (54). The percent variance captured by each coordinate was calculated by dividing each eigenvalue by the sum of the absolute value of all eigenvalues. Cross-species and cross-strain comparisons of metabolite production and consumption were made with the UpSetR package in R (55).

### Data availability.

Raw metabolomics files are publicly available on the MetaboLights database (study MTBLS2105; http://www.ebi.ac.uk/metabolights/MTBLS2105) (56). All processed metabolomics data, calculated fold changes, and raw and adjusted *P* values are available in the supplemental material (Data Sets S1 and S2). Data and code can additionally be found at https://github.com/lauradunphy/metabolomicsSCFM.

10.1128/mSystems.00480-21.6 DATA SET S2 Metabolomics metadata and raw and normalized peak areas for all samples. Download Data Set S2, CSV file, 3.0 MB. Copyright © 2021 Dunphy et al. 2021 Dunphy et al. https://creativecommons.org/licenses/by/4.0/ This content is distributed under the terms of the Creative Commons Attribution 4.0 International license.
