# Supplementary figures and images for: Untargeted Metabolomics Reveals Species-Specific Metabolite Production and Shared Nutrient Consumption by Pseudomonas aeruginosa and Staphylococcus aureus
Source: mSystems. 2021 Jun 22;6(3):e00480-21. doi: 10.1128/mSystems.00480-21 (PMC8269234; doi:10.1128/mSystems.00480-21)

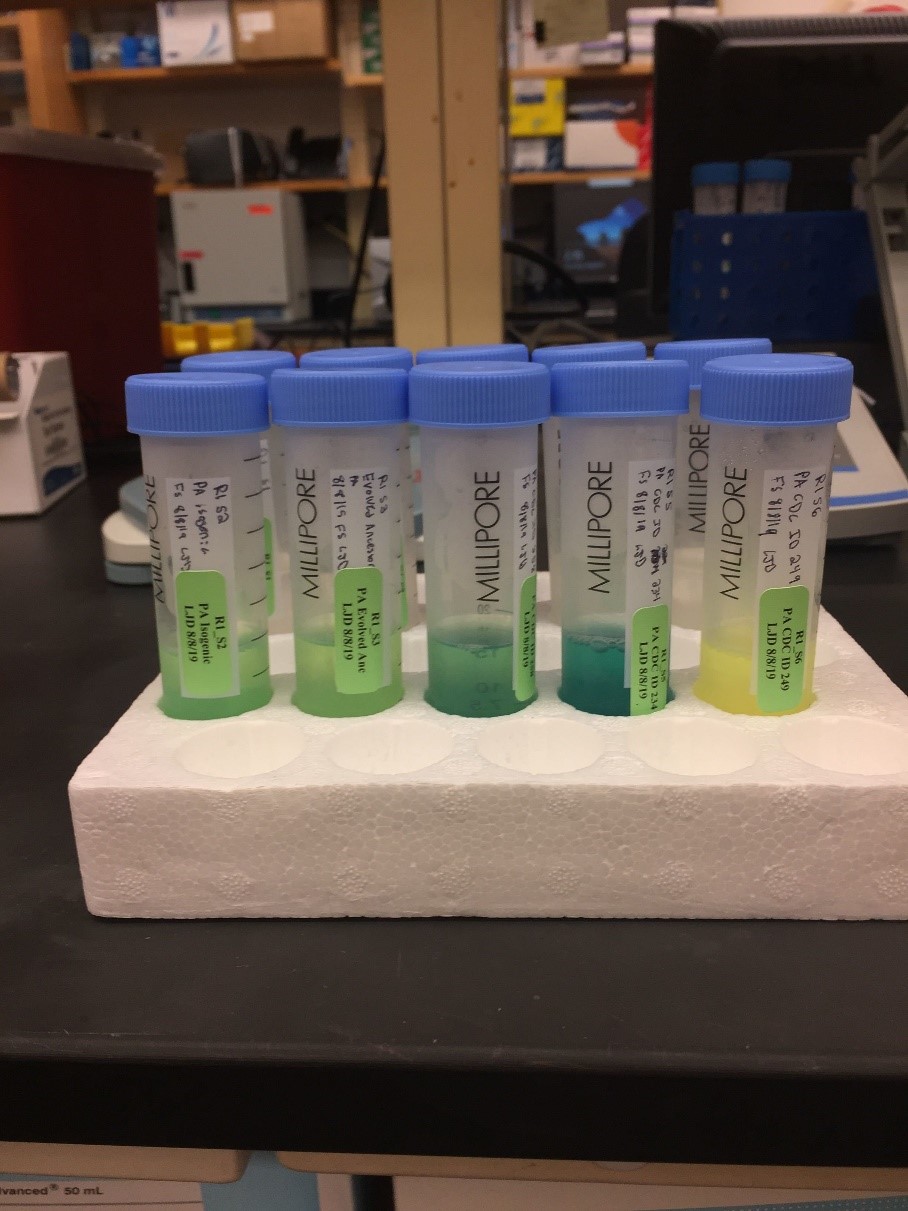

Supplement: FIG S1 [file msystems.00480-21-sf001.jpg]

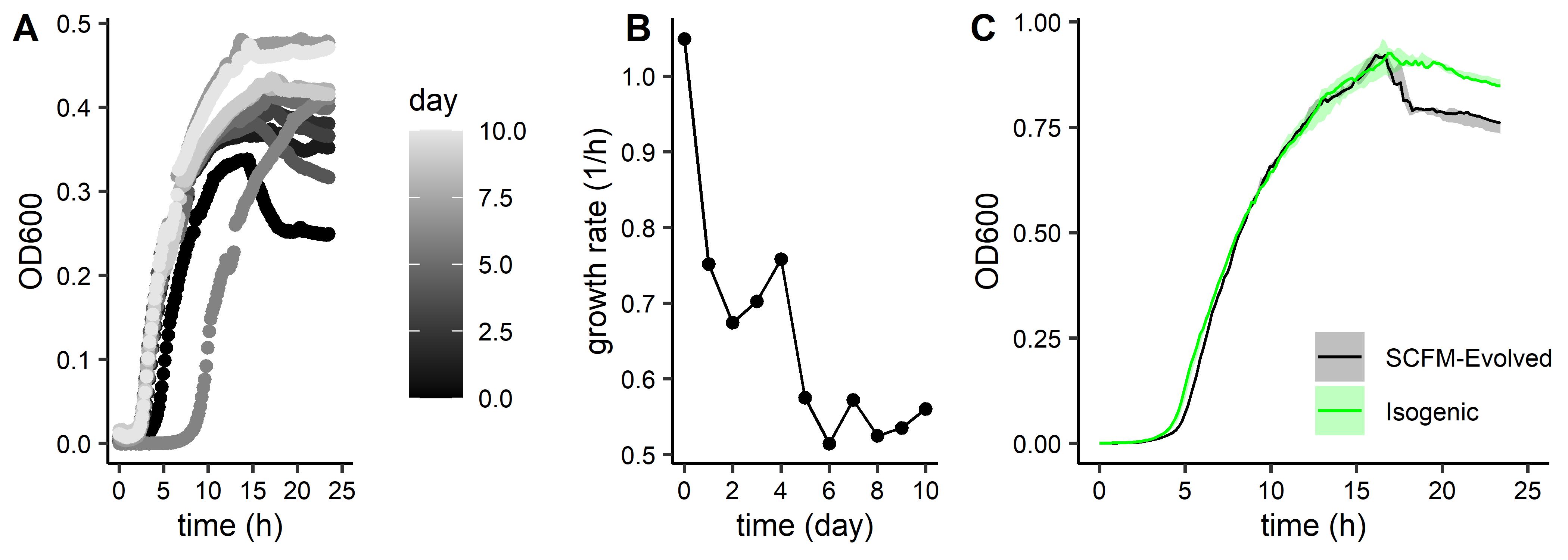

Supplement: FIG S2 [file msystems.00480-21-sf002.jpg]

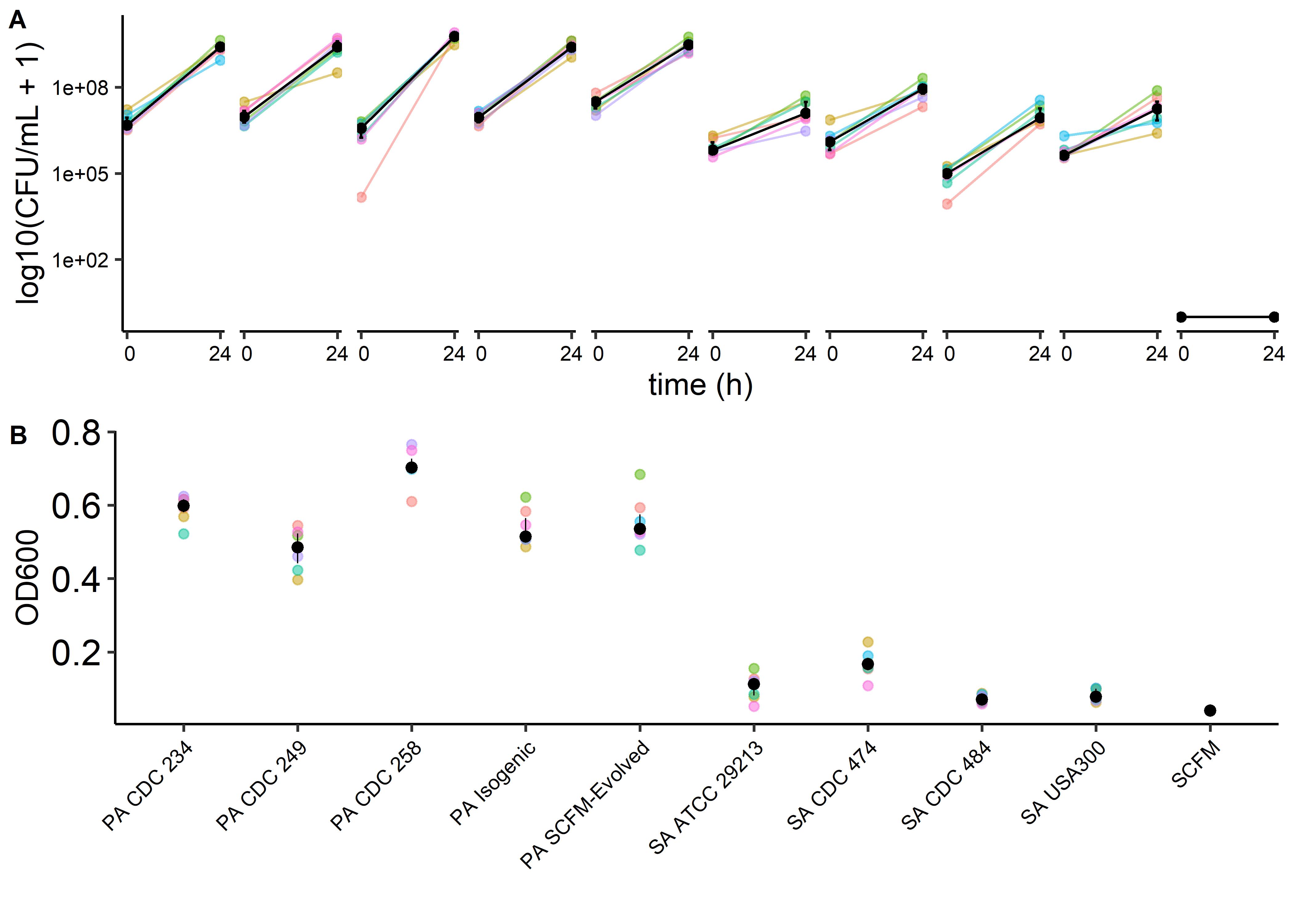

Supplement: FIG S3 [file msystems.00480-21-sf003.jpg]

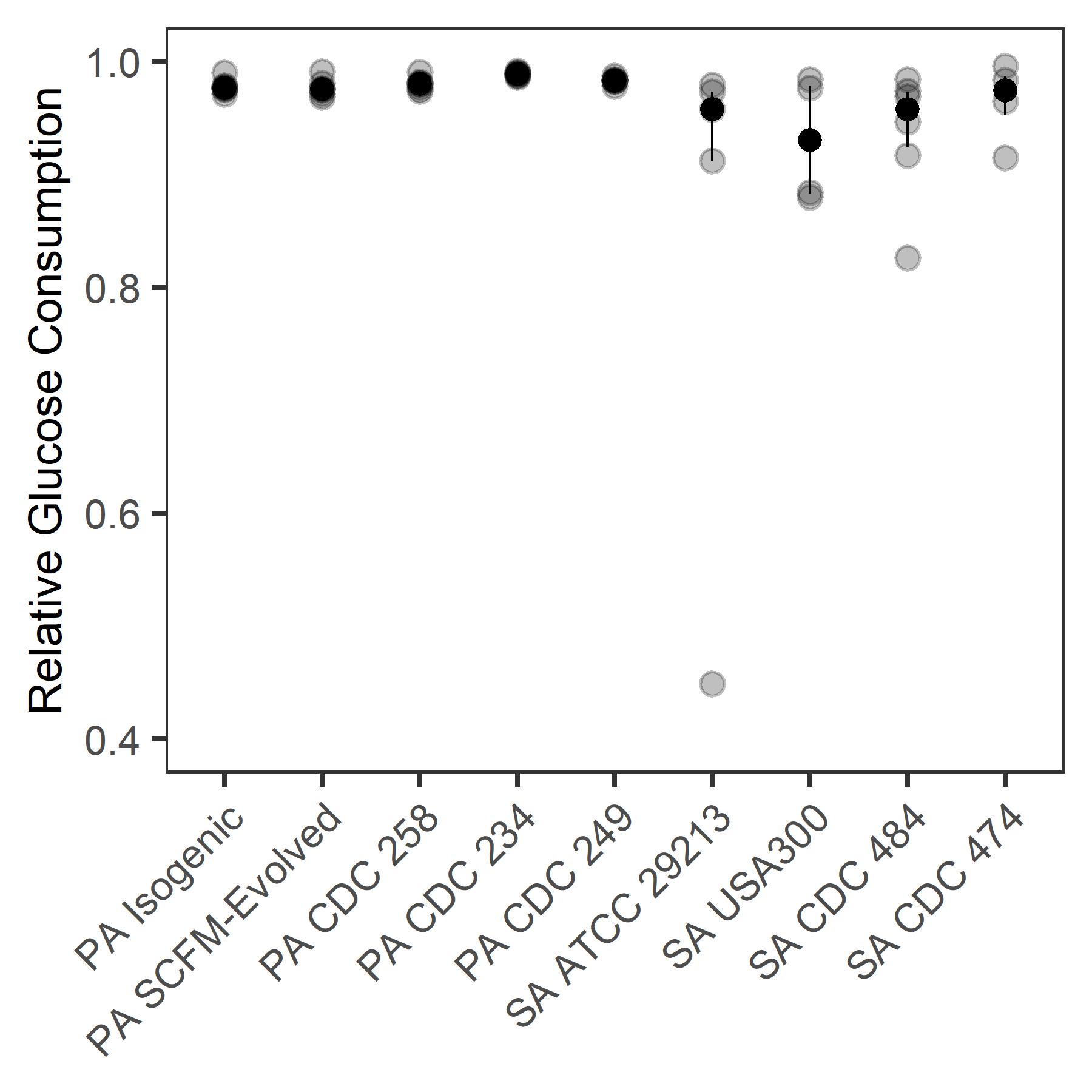

Supplement: FIG S4 [file msystems.00480-21-sf004.jpg]
